# CK2α通过PI3K/Akt/GSK-3β信号通路调控肺腺癌A549细胞的侵袭及迁移

**DOI:** 10.3779/j.issn.1009-3419.2017.04.11

**Published:** 2017-04-20

**Authors:** 爱兵 吴, 明春 黎, 宗炯 麦, 姝君 李, 志雄 杨

**Affiliations:** 1 524000 湛江，广东医科大学附属医院肿瘤中心 Department of Oncology, Affiliated Hospital of Guangdong Medical University, Zhanjiang 524000, China; 2 341000 赣州，赣南医学院附属医院肿瘤科 Department of Oncology, Af?liated Hospital of Gannan Medical University, Ganzhou 341000, China

**Keywords:** 肺肿瘤, 酪蛋白激酶CK2α, PI3K/Akt/GSK-3β信号通路, 上皮-间充质转化, 侵袭, 迁移, Lung neoplasms, CK2α, PI3K/Akt/GSK-3β signaling pathway, Mesenchymal-to-epithelial transition, Invasion, Migration

## Abstract

**背景与目的:**

肺癌已成为全球癌症死亡的首要原因，而侵袭和转移是导致肿瘤死亡的主要原因之一，蛋白激酶CK2是一种高度保守信使非依赖性丝氨酸苏氨酸蛋白激酶，其在各种肿瘤中高表达。本研究旨在探讨下调*CK2α*基因表达对肺腺癌A549细胞侵袭迁移的影响以及可能的机制。

**方法:**

构建pSilencer^TM^ 4.1-shCK2α-eGFP慢病毒表达载体，建立稳定干扰CK2α表达的A549细胞株。利用Transwell和Boyden小室实验检测干扰CK2α表达前后A549细胞的侵袭及迁移的能力。Western blot检测PI3K/Akt信号通路和上皮-间充质转化（mesenchymal-to-epithelial transition, EMT）相关蛋白的表达。

**结果:**

与对照组相比，干扰CK2α表达后肺腺癌A549细胞的侵袭及迁移能力明显下降，p-PTEN、Akt、p-Akt^473^、p-Akt^308^、p-PDK1、p-c-Raf、p-GSK-3β蛋白明显下调，PTEN蛋白表达水平显著上调。上皮-间充质转化的相关蛋白E-cadherin蛋白表达水平显著上调，而Vimentin、β-catenin、Snail蛋白表达水平显著下调，与侵袭转移相关蛋白的MMP2、MMP9表达水平显著下调。

**结论:**

CK2α可能通过PI3K/Akt/GSK-3β/Snail信号通路来调控上皮-间充质转化参与肺腺癌A549细胞的侵袭及迁移。

## Abstract (en)

**Background and objective:**

Lung cancer is the leading cancer-related death worldwide. Patients with lung cancer mainly died of tumor metastasis and invasion. Protein kinase CK2 is an ubiquitous serine/threonine protein kinase and is frequently upregulated in various human tumors. This study aims to explore the effect and molecular mechanism of the invasion and migration of lung adenocarcinoma A549 cells after knock-down of CK2α expression.

**Methods:**

The pSilencer^TM^ 4.1-siCK2α-eGFP of lentiviral-mediated shRNA was constructed. The expression of CK2α was knock-downed, and a stable A549 cell line was established. The invasion and migration of A549 cell line was detected through Transwell and Boyden chamber assays. The protein expression of the PI3K/Akt signaling pathway and mesenchymal-to-epithelial transition (EMT) was evaluated using Western blot analysis.

**Results:**

The invasion and migration of A549 cells were significantly inhibited after the knockdown of CK2α expression compared with that in the control group. p-PTEN, Akt, p-Akt^473^, p-Akt^308^, p-PDK1, p-c-Raf, and p-GSK-3β were significantly downregulated, whereas PTEN was upregulated. Moreover, vimentin, β-catenin, Snail, MMP2, and MMP9 were significantly downregulated after reducing the CK2α expression.

**Conclusion:**

CK2α might regulate the invasion and migration of A549 cells through the PI3K/Akt/GSK-3β/Snail signaling pathway, which controls EMT in lung adenocarcinoma.

肺癌已成为全球癌症相关性死亡的首要原因之一，每年肺癌的新发病例人数与死亡病例人数都在逐渐增加，每年约有160万人死于肺癌^[1]^。而非小细胞肺癌（non-small cell lung cancer, NSCLC）在所有肺癌类型中所占比例将近85%，是最常见的发病类型，其中肺腺癌是主要的病理类型。超过57%的患者被发现时已被诊断为肺癌晚期。已有远处转移的晚期患者，5年生存率 < 5%，中位生存时间 < 12个月。远处转移是导致肺癌患者的死亡原因之一，因此研究NSCLC的侵袭转移的机制具有重要意义。

蛋白激酶CK2，曾称为酪蛋白激酶2或Ⅱ（casein kinase 2 or Ⅱ），是一种高度保守信使非依赖性丝氨酸苏氨酸蛋白激酶。它是由两个催化亚基（α/α’）和两个调节亚基β构成的不均一四聚体^[2, 3]^。CK2是一种多功能的蛋白激酶，其磷酸化底物具有多样性，迄今已发现它有300多种底物^[4]^，涉及到细胞的生长、增殖、凋亡、分化、侵袭和转移^[5-7]^。研究发现CK2在肾癌^[8]^、肺癌^[9]^、头颈部癌^[10]^、前列腺癌^[11]^、乳腺癌^[12]^、大肠癌^[13]^、白血病^[14]^等多种肿瘤中高表达。CK2通过磷酸化它的底物促进肿瘤细胞的增殖、侵袭和转移，而抑制细胞凋亡，在肿瘤的发生发展中起重要作用。Kim等^[15]^发现在肺癌细胞株A549中，CK2抑制剂CX-4945能抑制Smad2/3、Twist、Snail、Akt等调节上皮-间充质转化（mesenchymal-to-epithelial transition, EMT）的整个过程，可以抑制A549细胞的迁移和侵袭并伴有MMP-2和MMP9的下调。Liu等^[16]^发现CK2α靶向抑癌基因BRMS1核导出和降解来促进肺癌侵袭转移。但是CK2α在NSCLC侵袭转移的机制还是不清楚。

本研究利用RNA干扰技术，干扰肺腺癌A549细胞中CK2α的表达，分析对肺癌A549细胞的侵袭迁移能力的影响以及机制的初步研究。

## 1 材料和方法

### 1.1 一般材料

pSilencer^TM^ 4.1-shRNA载体购于Ambion公司，限制性内切酶*Bam*H Ⅰ和限制性内切酶*Hin*d Ⅲ（Fermentans公司），DH5a感受态细胞购于Auragene公司，琼脂糖凝胶DNA回收试剂盒购于TIAGEN公司，DNA Ladder Marker购自日本Takara公司，脂质体Lipofectamine^TM^ 2000、G418试剂盒，RPMI-1640为美国Invitrogen公司产品。胎牛血清、蛋白裂解液、PVDF膜购自华奇盛公司。PTEN、p-PTEN、Akt、p-Akt^473^、p-Akt^308^、p-GSK-3β、p-PDK1、p-c-Raf、E-cadherin、Vimentin、β-catenin、Snail、MMP2、MMP9、β-actin抗体购于Cell Signaling Technology公司，二抗购于中杉金桥公司，Transwell小室购于Corning公司。

### 1.2 细胞培养

人肺腺癌细胞株A549为贴壁细胞，为广东医科大学附属医院临床科研中心保存，培养于含10%胎牛血清和1%的青-链双抗的RPMI-1640培养基中，置于5%CO_2_、37 ℃恒温细胞培养箱中培养，2 d-3 d传代，细胞生长状态良好时用于实验。

### 1.3 载体构建及转染细胞

由长沙艾佳生物技术有限公司设计3个靶点siRNA序列，选择最有效CK2α的siRNA片段，具体的序列为：shCK2α1028：Sense 5’-GATCCCAGAAGATTTATATGACTATTCAAGAGATAGTCATATAAATCTTCTGA-3’，shCK2α1028 Antisense：5’-AGCTTCAGAAGATTTATATGACTATCTCTTGAATAGTCATATAAATCTTCTGG-3’。在T4 DNA连接酶催化下将载体与退火后的互补引物连接，重组体转化大肠杆菌DH5α，利用氨苄霉素进行筛选，挑选阳性克隆进行测序，将阳性克隆在大肠杆菌DH5α中扩增，提取质粒。用Lipofectamine^TM^ 2000脂质体介导转染重组质粒，包装病毒，感染A549细胞，进行单克隆挑选，利用qPCR和Western blot鉴定干扰效果，CK2α引物Sense：5’-CAAACTGCTGCGATATGACCAC-3’；Antisense：5-GGCACTGAAGAAATCCCTGAC-3，建立稳定干扰CK2α的A549细胞株。

### 1.4 细胞迁移实验

取生长状态良好的培养细胞，用PBS液洗3次，0.25%胰酶消化细胞制成单细胞悬液。调整细胞浓度为1×10^6^/mL。在24孔板内加入500 μL含10%胎牛血清的培养液。小室内加入100 μL（含1×10^5^个细胞）的无血清单细胞悬液，12 h-14 h后收小室。利用棉签擦拭小室内的细胞，加PBS液清洗。将小室放入500 μL甲醇的24孔板内，固定约15 min。取出小室，擦干小室内的甲醇，浸入苏木精染液中染色20 min，在空气中风干。400倍显微镜下随机5个视野观察细胞，计数，实验重复3次。

### 1.5 细胞侵袭实验

利用4 ℃预冷的无血清培养基稀释Matrigel（按1:8稀释），在chamber上室底部中央垂直加入100 μL稀释后的Matrigel，37 ℃温育4 h-5 h使其干成胶状，取对数生长期细胞、胰酶消化、加培养基终止消化，离心2 min 800 rpm，用无血清培养基重悬，调整细胞加100 μL无血清细胞悬液于小内室，在24孔板下室加入500 μL含20%FBS的培养基然培养箱孵育16 h。收集小室，利用棉签擦拭小室内细胞，在甲醇内室温下固定15 min，自然晾干，用苏木精应用染液染色20 min，在PBS液清洗3遍，空气风干。400倍显微镜下随即选取5个视野观察细胞，记数。实验重复3次。

### 1.6 Western blot实验

取对数生长期细胞，提取蛋白，测蛋白浓度，配制不同浓度的SDS聚丙烯酰胺凝胶，并加入每泳道40 μg蛋白进行电泳。电泳结束后，转移蛋白至PVDF上。3%牛血清白蛋白封闭后，分别加入PTEN、p-PTEN、Akt、p-Akt^473^、p-Akt^308^、p-GSK-3β、p-PDK1、p-c-Raf、E-cadherin、Vimentin、β-catenin、Snail、MMP2、MMP9和β-actin抗体进行孵育。然后用二抗进行孵育，用奥德赛条带扫描仪扫描蛋白条带。

### 1.7 统计学方法

采用SPSS 13.0统计软件处理数据，各指标以均值±标准差（Mean±SD）来表示，多组采用单因素方差分析（*One-Way ANOVA*），细胞间多重比较采用*LSD*检验。*P* < 0.05为差异有统计学意义。

## 2 结果

### 2.1 siRNA干扰CK2α表达的效率

为检测CK2α表达干扰效率，利用荧光定量RT-PCR鉴定干扰后单克隆细胞中CK2α的表达，结果显示：与空载对照（Con）组和正常对照（NC）组相比，sh870、sh1028组干扰率最高，其干扰效率均大于80.0%，结果显示各细胞中CK2α的表达有显著差异（*F*=46.900, *P* < 0.001）（图 1A）。为进一步检测CK2α表达干扰效率，利用Western blot检测干扰后CK2α蛋白的表达，以β-actin蛋白为内参，根据各条带的CK2α灰度值与β-actin灰度值比率计算各单克隆细胞中CK2α的表达有显著差异（*F*=339.528, *P* < 0.001）（图 1B），其中sh870和sh1028组的干扰效率最高，选取这两株细胞进行实验。

**1:**
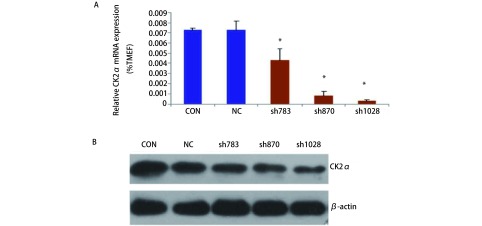
siRNA干扰CK2*α*表达的效率A：荧光定量PCR检测CK2*α*的表达水平；B：Western blot检测CK2*α*的表达水平。*：*P* < 0.05 Analyzing the efficiency of down-regulated CK2*α* with siRNA. A: Testing the expression of CK2*α* by Real-time PCR; B: Testing the expression of CK2*α* by Western blot. *: *P* < 0.05

### 2.2 *CK2α*基因干扰对A549细胞体外迁移和侵袭能力的影响

为检测细胞的迁移能力，采用Transwell小室实验检测CK2α表达干扰后四组细胞体外迁移能力的变化，结果发现与Con组（333.67±31.565）和NC组（334.00±31.000）细胞相比，sh870（88.33±6.807）、sh1028组（96.00±10.817）穿过膜的细胞数明显减少（*P* < 0.05，图 2A）。为进一步检测细胞的侵袭能力，采用Boyden小室实验的方法检测CK2α表达干扰后细胞体外侵袭能力的变化，结果显示，与Con组（134. 67±10.066）和NC组（134.67±7.638）细胞相比，sh870（62.00±6.000）与sh1028（61.33±5.686）细胞穿过基质胶的细胞数明显减少（*P* < 0.05，图 2B）。这都提示干扰CK2α表达后，A549细胞体外迁移侵袭能力明显降低。

**2:**
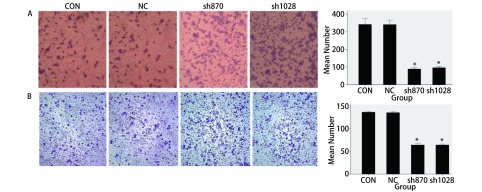
*CK2α*基因干扰对A549细胞体外迁移和侵袭能力的影响。A：Transwell实验检测A549细胞的迁移能力；B：Boyden小室实验检A549测细胞的侵袭能力。* *P* < 0.05 Effects of knock-down CK2*α* expression Comment on invasion and migration of A549 cell line. A: Analyzing the migration of A549 cell line via Transwell assay; B: Analyzing the invasion of A549 cell line via Boyden assay. *P < 0.05

### 2.3 检测干扰CK2α表达前后EMT相关蛋白及MMP2和MMP9蛋白表达水平

为进一步证明CK2α参与肿瘤的侵袭迁移，我们利用Western blot检测干扰CK2α表达后四组细胞中EMT相关蛋白及MMP2和MMP9蛋白表达水平。结果显示，上皮细胞的分子标记E-cadherin在sh870和sh1028组中表达显著高于Con和NC组（*P* < 0.01，图 3）；相反，间质细胞的分子标记物Vimentin、β-catenin，转录因子Snail在sh870和sh1028组中的表达显著低于NC组和Con组（*P* < 0.01，图 3）。我们进一步利用Western blot检测MMP2、MMP9的表达，结果显示，MMP2、MMP9在sh870和sh1028组中的表达显著低于NC组和Con组（*P* < 0.01，图 3）。

**3:**
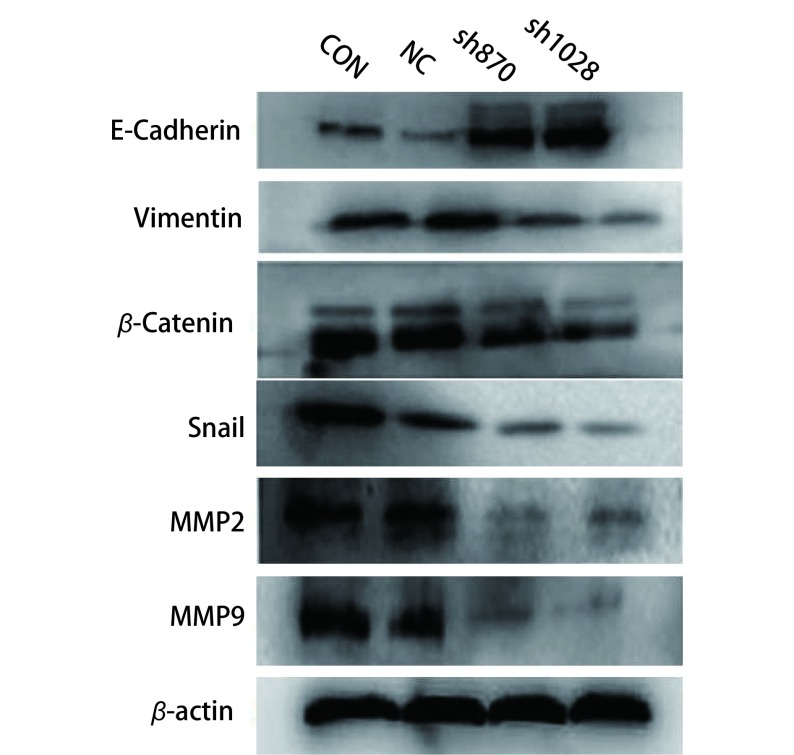
Western blot检测上皮细胞-间充质转化和转移相关蛋白的表达水平 Testing proteins expression of mesenchymal-to-epithelial transition and relative metastasis by Western blot

实验结果表明，干扰CK2α表达可抑制EMT的发生，下调MMP2、MMP9的表达，抑制细胞的侵袭迁移。

### 2.4 Western blot检测干扰CK2α表达前后PI3K/Akt信号通路相关蛋白表达水平

为探讨CK2α可能调控EMT的机制，我们利用Western blot检测干扰CK2α表达前后PI3K/Akt信号通路中蛋白的表达水平，结果显示：抑癌基因*PTEN*在sh870和sh1028组中表达显著高于Con和NC组，而*Akt*、*p*-*Akt^473^*、*p*-*Akt^308^*、*p*-*PTEN* *p*-*PDK1*、*p*-*c*-*Raf*、*p*-*GSK*-*3β*基因在sh870和sh1028组中表达均显著下调（图 4）。

**4:**
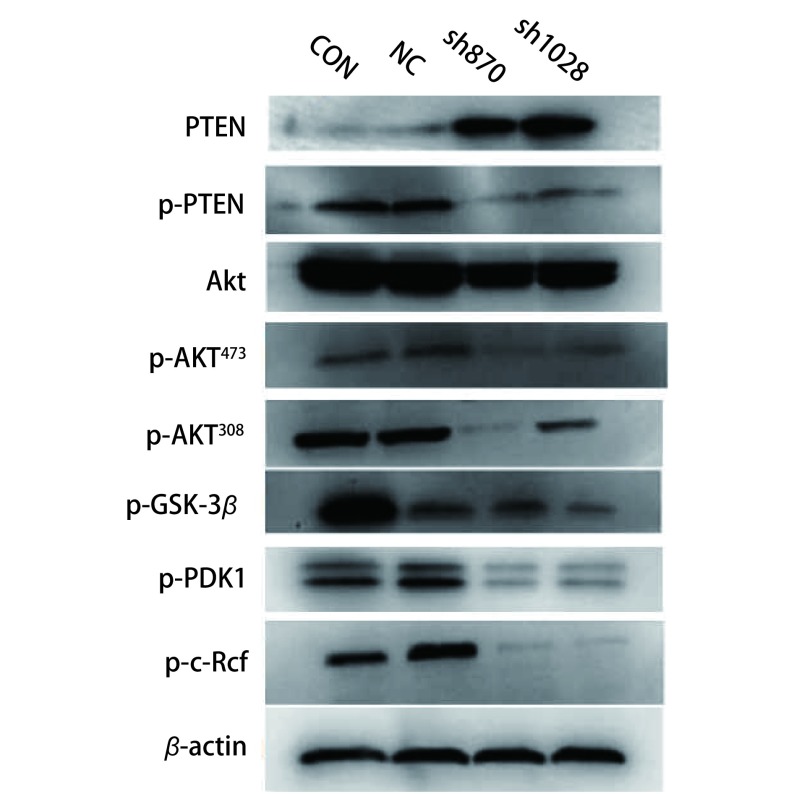
Western blot检测PI3K/Akt信号通路相关蛋白表达水平 Testing proteins expression of PI3K/Akt signaling pathway by Western blot

实验结果提示，干扰CK2α表达可上调PTEN，减少PTEN的磷酸化及Akt的活化，从而阻止所有由Akt调控的下游信号传导事件，即使Akt、p-Akt^473^、p-Akt^308^、p-GSK-3β、p-PDK1、p-c-Raf蛋白表达下调。从而表明，干扰CK2α表达可抑制Akt的活化，抑制细胞的侵袭和转移。

## 3 讨论

肺癌侵袭转移是一个复杂过程，其涉及多个癌基因与抑癌基因和多条信号通路的调控。虽然近年来肺癌的诊断和治疗水平有很大的提高，但是总的生存时间没有多大的提高，导致肺癌治疗失败的主要原因之一就是侵袭转移。因此探究肺癌细胞侵袭和转移生物学行为，对于指导肺癌的诊断、治疗及预后评判十分重要。CK2是多功能的蛋白激酶，其磷酸化底物具有多样性，涉及到细胞的生长、增殖、分化、凋亡。近年来，有研究^[15, 16]^显示CK2参与肿瘤的侵袭转移，但在肺癌中机制还不是很清楚。

我们利用siRNA技术，干扰肺腺癌A549细胞中CK2α的表达，结果发现干扰CK2α的表达后，肺腺癌A549细胞的侵袭迁移能力显著降低，同时检测到MMP2和MMP9蛋白也明显下调，这都提示CK2α参与肺腺癌的侵袭迁移的过程。但是具体机制不清。上皮细胞-间充质细胞转化，是具有极性的上皮细胞转换成具有活动能力、能够在细胞基质间自由移动的间质细胞的过程。越来越多的证据证明EMT在肿瘤的侵袭、转移过程中起着重要作用^[17, 18]^。我们利用Western blot检测干扰CK2α的表达后EMT相关蛋白的表达，发现上皮分子标记物E-cadherin蛋白上调，而间质细胞的分子标记物Vimentin、β-catenin及转录因子Snail蛋白下调。这提示CK2α通过EMT来参与肺腺癌的侵袭迁移。但是CK2α通过哪条信号通路调控EMT来参与肺腺癌的侵袭迁移?

PI3K/Akt信号通路是细胞内重要的信号转导途径之一，参与很多重要的生物学过程的调控，其通过影响下游多种效应分子的活化状态，在细胞内发挥着抑制凋亡、促进增殖的关键作用。几年来研究发现，CK2与PI3K/Akt信号通路具有相互作用。Ryu等^[19]^发现在人类前列腺癌LNCaP细胞株中，CK2抑制剂CX4945抗雄激素受体活性，其通过抑制Akt-survivin信号通路发挥抗肿瘤作用。Maira等^[20]^发现CK2能磷酸化并上调Akt/PKB，产生抗凋亡，促进肿瘤的发生。Shehata等^[21]^发现CK2抑制剂在慢性淋巴细胞白血病细胞中减少了PTEN和Akt的磷酸化，促进了肿瘤细胞的凋亡，产生抗肿瘤作用。近年来有研究表明，PI3K/Akt信号通路可通过调控EMT，对肿瘤起着促进侵袭转移。Grille等^[22]^发现PI3K/AKT信号通路参与诱导鳞癌细胞EMT的发生，促进肿瘤细胞的侵袭性和转移性。PI3K/AKT信号通路是怎样调控EMT的？

GSK-3β是一种由丝氨酸/苏氨酸组成的多功能激酶，在调节糖原代谢起关键作用。它是PI3K/AKT信号通路下游基因，可磷酸化Snail转录因子调控EMT。Li等^[23]^发现在肺癌中OLA1通过GSK3β/Snail/E-cadherin调控EMT，从而调节肺癌的侵袭转移。同样在乳腺癌和胃癌中也发现PI3K/AKT/GSK3β信号通路调控EMT^[24, 25]^。因此可以认为，PI3K/AKT信号通路下游基因GSK-3β磷酸化Snail转录因子调控EMT，从而参与肿瘤的侵袭转移。我们干扰肺腺癌A549细胞中CK2α的表达后，抑癌基因PTEN表达升高，而*Akt*、*p*-*Akt^473^*、*p*-*Akt^308^*、*p*-*PTEN* *p*-*PDK1*、*p*-*c*-*Raf*、*p*-*GSK*-*3β*基因表达均显著下调，同时EMT相关蛋白E-cadherin蛋白上调，而间质细胞的分子标记物Vimentin、β-catenin及转录因子Snail蛋白下调。我们认为CK2α可能是通过PI3K/AKT/GSK3β信号通路调控Snail转录因子来调节EMT，从而参与肺腺癌A549细胞侵袭转移。

综上所述，PI3K/Akt信号通路与EMT存在复杂的调控关系，我们认为CK2α可能是通过PI3K/AKT/GSK3β信号通路调控Snail转录因子来调节EMT，因此，CK2α是通过PI3K/AKT/GSK3β/Snail信号通路调控肺腺癌A549细胞侵袭转移。是否CK2α可能还通过其他信号通路对EMT的调控，还需要更多实验去验证。
